# Evaluation of the knowledge regarding vitamin D, and sunscreen use of female adolescents in Iran

**DOI:** 10.1186/s12889-021-12133-5

**Published:** 2021-11-10

**Authors:** Afsane Bahrami, Zahra Farjami, Gordon A. Ferns, Parichehr Hanachi, Majid Ghayour Mobarhan

**Affiliations:** 1 Clinical Research Development Unit of Akbar Hospital, Faculty of Medicine, Mashhad University of Medical Sciences, Mashhad, Iran; 2 Clinical Research Development Unit, Imam Reza Hospital, Faculty of Medicine, Mashhad University of Medical Sciences, Mashhad, Iran; 3 Metabolic Syndrome Research Center, Mashhad University of Medical Sciences, Mashhad, Iran; 4 Brighton & Sussex Medical School, Division of Medical Education, Falmer, Brighton, Sussex, BN1 9PH UK; 5 Department of Biology, Biochemistry Unit, Al Zahra University, Tehran, IR Iran

**Keywords:** Vitamin D, Sun-screening agents, Knowledge, Behavior, Diet

## Abstract

**Background:**

Vitamin D (Vit D) deficiency/insufficiency is an important risk factor for several chronic conditions. We aimed to evaluate the knowledge and behavior of female adolescents with respect to the association between sunlight exposure, sunscreen use, and Vit D status.

**Methods:**

This cross-sectional survey was performed in northeastern Iran, among 940 female adolescents in January 2015. Each subject completed a questionnaire containing items about demographic characteristics, knowledge about Vit D and their use of sunscreen. Serum Vit D levels were measured using an electrochemiluminescence method and dietary intake of Vit D was assessed using a Food Frequency Questionnaire. Statistical analyses were conducted using SPSS software. A *P* value < 0.05 was considered statistically significant.

**Results:**

Few of the participants were aware of the biological functions of Vit D (8.8%), the causes of Vit D deficiency (16.7%), and the sources of Vit D (9.3%). Less than half of the participants used sunscreen during the day. The serum levels of Vit D in subjects who used sunscreen were significantly lower than those who did not (*p* = 0.004). However, there was no significant association between their knowledge about Vit D and serum Vit D, or dietary intake of Vit D.

**Conclusion:**

There appears to be a lack of coherence between lifestyle, behavior and knowledge that may affect the Vit D status of adolescent girls in northeastern Iran. This information provides a basis for developing public health planning (workshops or training at the college level) for the prevention of Vit D deficiency especially in adolescent girls.

**Supplementary Information:**

The online version contains supplementary material available at 10.1186/s12889-021-12133-5.

## Background

Vitamin D (Vit D) has an important role in bone health, calcium homeostasis and immune regulation [1, 2]. Furthermore, Vit D deficiency may enhance the risk of cancer, diabetes mellitus types 1 and 2, tuberculosis and cardiovascular disease [3]. Deficiency of Vit D is also positively associated with the morbidity from several conditions, that include: cardiovascular disease, diabetes, obesity and autoimmune diseases [4–7]. Vit D deficiency is common in many developing countries [8]. Whilst several chronic diseases are associated with Vit D insufficiency, patients with adequate levels of Vit D have been shown to have better health outcomes [9]. In Asian countries, the risk of Vit D deficiency may be underestimated as it may be taken for granted that individuals receive sunshine throughout the year. However, Vit D status has been shown to be compromised in Middle Eastern countries despite the intense prevailing sunshine [10]. The prevalence of Vit D deficiency in China, Turkey, India, Iran, and Saudi Arabia, has been estimated to be 30–93% over the last 2 decades [11].

Several factors may affect the prevalence of Vit D deficiency, including latitude, duration of sun exposure, clothing, season and skin pigmentation [12]. Fair skin is favored among Asian women; in addition, they believe that sunlight affects skin wrinkling and skin pigmentation. Deficiency of Vit D is very common in India, and Vit D deficiency is prevalent in tropical countries overall [13].

A recent study demonstrated a high frequency of Vit D insufficiency among Iranians [11] and this is also true for the neighboring countries despite their ample sunlight [14] with a prevalence of reaching 85% in some regions [15]. The time spent outside of the home, and exposure to sunlight, have fallen in Iran due to urbanization, changes in lifestyle and, technology, and rising temperatures [16–18]. In addition, there have been changes in dietary habits, especially in the younger generations. Iranian women often cover their body, shielding their skin from sunlight, and this may also contribute to Vit D deficiency [19]. On the other hand, exposure to ultraviolet (UV) radiation in sunlight may increase the risk of skin cancer; the incidence rate of this cancer is 10.13 to 28.1 per 100,000 people In Iranian population [20, 21] which is lower than in Australia, Norway and Denmark (33.6, 29.6 and 27.6 per 100,000 people respectively) [22]. Another source of Vit D is fortification or supplementation [23]. Government recommendations in Iran have suggested using 50,000 IU vitamin D3 supplement per month for adult (19–59 years) in order to prevent vitamin D deficiency [24].

The knowledge, and behaviors of the population with respect to Vit D, may play an important role in the prevention of chronic disease. In the Iranian population, young girls are at a particularly high risk of Vit D deficiency [19], due to their tendency to shield themselves from exposure to the sun and cover their face, hands with protective sunscreen creams.

There is evidence for a lack of basic awareness of the role and importance of Vit D globally [25–27]. There have been no reports with respect to the knowledge of the importance of Vit D amongst adolescent females in Iran. The aim of this study was to investigate the behavior, and knowledge of Iranian adolescent girls with respect to sunscreen, Vit D and its importance to health.

## Methods

### Population study

This cross-sectional study among adolescent girls was performed in Mashhad and Sabzevar, two cities in Iran as described previously [28, 29]. Briefly, the population samples were recruited from 6 different geographic areas in 2 cities in January 2015, using a multistage cluster randomized sampling approach. Four high schools from each of the 6 geographic areas were chosen, and 1 class from each grade (3 classrooms from each school) was randomly selected for inclusion. In each classroom, approximately 15 students were included. Schools, classes and students were recruited using computer-generated random numbers. Inclusion criteria were: an age of between 12 and 19 years, single, and apparent healthy status. We excluded those with any acute or chronic diseases, as well as girls who were receiving anti-inflammatory, antidepressant, antidiabetic or antiobesity drugs, Vit D or calcium supplement use, and hormone therapy within the previous 6 months. A total of 1026 subjects were initially approached, of whom 956 met the inclusion criteria. The Ethics Committee of the Mashhad University Medical School approved the study (IR.MUMS.fm. REC.1395.12). Written consent was obtained from students and their parents.

### Data collection instruments

The questionnaire comprised of 3 sections (Supplementary File 1). Section 1 comprised demographic information of the participants such as family members, type of house and rooms they inhabited, the occupation of their parents and their educational attainment using a standard questionnaire which was previously validated in the PIRLS for Iran [30, 31]. Section 2 was concerning behavior towards the usage of sunscreen. This section including items exploring the frequency, seasons, amount and the location as well as sun protection factor (SPF) of sunscreen usage. Section 3 was designed to evaluate their knowledge on Vit D, and comprised 5 questions: on the role of Vit D in the body, the main sources of Vit D, factors that cause Vit D deficiency and related diseases such as osteoporosis; the questionnaire was previously developed by Kung et al., in Hong Kong and was translated into Persian [25]. The questionnaire was completed in the presence of the researcher to prevent the use of the internet, or mobile phones to access information. We assessed test-retest reliability of the questionnaire in a sample of 20 individuals, with a 2 weeks interval between the tests. Test-retest reliability and validity of this questionnaire (range of intra-class correlation coefficient for three sections is 0.73–0.96 and Cronbach’s Alpha was 0.71–0.88) was established for this population.

A validated food frequency questionnaire (FFQ) was used to assess dietary intakes, and was validated for the Iranian population in a previous study [32–34]. The questionnaire included 65 food items with 5 frequency categories (frequency of intake: daily, weekly, monthly, rarely, and never) for each food item and portion size. To calculate energy and nutrient intakes, the reported portion size in FFQ were converted to grams by using household measures and subsequently were entered into the Nutritionist IV software for analysis.

### Evaluation of vitamin D levels

Fasting blood samples were obtained early in the morning following a 12 h overnight fast. Blood samples were immediately centrifuged to separate serum and samples were stored at -80^o^ C at the reference laboratory. An electrochemiluminescence method (ECL, Roche, Switzerland) was used for the measurement of serum 25-hydroxy vitamin D (25OHD) concentrations.

### Data analysis

A Kolmogrov–Smirnov test was used to assess the normality of the distribution of variables. Descriptive statistics such as means and standard deviations (for normally distributed data) or median and interquartile range (IQR) (for non-normally distributed data) as well as frequency and percentages are provided. For normally distributed variables, independent sample T-test or one-way ANOVA test and for non-normally distributed variables, the Mann-Whitney test or Kruskal-Wallis test were used. A *P* value< 0.05 was considered statistically significant. Statistical analyses were performed by SPSS version 16.0 (SPSS Inc., Chicago, Ill., USA).

## Results

The socio-demographic characteristics in relation to serum Vit D levels of the participants are shown in Table 1. The mean age of participants was 14.6 ± 1.5 (range: 12 to 18) years. Eight hundred and ninthly seven (95.4%) of them had reached the age of menstruation and puberty. Serum Vit D levels were not related to attainment of puberty, other family members sharing their room, or having an exclusive room, parental divorce, as well as mother/father occupations and educational attainment (*P* > 0.05). However, girls who were orphans had a significantly lower serum Vit D levels compared to other groups (*p* = 0.019).

**Table 1:** **Demographic and clinical characteristics of the participant (*N*** **= 940)**

***Variables***	Number (%)	Vitamin D level* (ng/ml)	P value**
**Puberty**			
Yes	897 (95.4)	6.8 (4.0–9.9)	0.68
No	43 (4.6)	6.5 (4.1–11.0)	
**Family members**			
2–4	447 (47.55)	6.7 (3.8–9.8)	0.247
5–7	458 (48.68)	6.6 (4.1–10.1)	
8–10	29 (3.11)	8.0 (4.7–10.4)	
> 11	6 (0.66)	9.1 (6.0–16.0)	
**Roommate**			
Father and mother	864 (91.98)	6.7 (4.0–10.2)	0.117
Father	9 (0.94)	5.4 (3.5–9.6)	
Mother	57 (6.04)	6.7 (3.7–9.0)	
Other family	10 (1.04)	8.4 (7.5–37.4)	
**Exclusive room**			
Yes	498 (52.95)	6.8 (4.2–10.5)	0.209
No	442 (47.05)	6.7 (3.8–9.4)	
**Parental death**			
Yes	40 (4.21)	4.3 (3.6–7.9)	**0.019**
No	900 (95.79)	6.7 (4.1–10.2)	
**Parental divorce**			
Yes	41 (4.51)	6.4 (4.5–10.1)	0.943
No	899 (95.49)	6.7 (4.0–10.1)	
**Paternal Occupation**			
Worker	320 (34.00)	6.9 (4.6–9.4)	0.363
Employee	169 (17.96)	6.7 (4.0–11.9)	
Tradesmen market	186 (19.79)	6.7 (3.6–10.3)	
Spiritual	7 (0.77)	8.1 (5.6–20.0)	
Other	254 (27.09)	6.5 (3.7–9.5)	
Deceased	4 (0.38)	8.1 (4.9–17.8)	
**Maternal Occupation**			
Worker	8 (0.85)	6.5 (3.6–9.1)	0.668
Employee	38 (4.08)	6.2 (3.0–10.1)	
Housewife	812 (86.35)	6.8 (4.0–10.2)	
other	77 (8.25)	6.5 (4.3–9.4)	
Deceased	5 (0.47)	4.5 (3.0–6.0)	
**Educational attainment of father (year)**			
0–9	372 (39.6)	6.5 (4.0–9.0)	0.103
10–12	438 (46.6)	6.9 (3.9–10.2)	
≥ 13	129 (13.8)	7.1 (4.3–15.9)	
**Educational attainment of mother (year)**			
0–9	448 (47.66)	5.2 (3.0–10.0)	0.168
10–12	362 (38.51)	6.6 (4.0–10.2)	
≥ 13	130 (13.83)	7.2 (4.5–10.4)	

*Vitamin D levels expressed as median (interquartile range)

**By using Mann-Whitney test or Kruskal-Wallis test

### Knowledge of the participants about Vit D

A significant number of participants (39.4%) stated that they were aware about the role of Vit D and few participants (8.8%) stated that they were completely aware about the role of Vit D. When asked about the causes of Vit D deficiency, 30.7% of participants were somewhat aware of these causes, whereas, 16.7% of participants were completely aware about the causes of deficiency of Vit D and 52.6% of participants did not know about the health impact of a reduction of Vit D status. Although, Vit D has been important role in bone health and osteoporosis, 74.5% of the participants did not know about the role of Vit D in osteoporosis. Overall, there was no significant association with the knowledge of the participants about Vit D and its serum levels and dietary intake (*P* > 0.05; Table 2).

**Table 2:** Knowledge of the participants about Vitamin D (N = 940)

Questions		Completely aware	Somewhat aware	Don’t know
**Role of vitamin D in the body** Correct answer*:	N (%)	83 (8.8%)	370 (39.4%)	487 (51.8%)
-bone health, calcium absorption	Vitamin D level (ng/ml)	6.8 (3.9–13.7)	6.4 (3.7–9.9)	7.0 (4.3–9.9)^NS^
-prevention of many chronic illnesses (i.e. cancer, diabetes, stroke, arthritis, and high blood pressure)	Vitamin D intake (μg)	3.0 ± 1.4	3.1 ± 1.8	3.2 ± 1.8 NS
**Cause of vitamin D deficiency** *Correct answer:*	N (%)	157 (16.7%)	289 (30.7%)	494 (52.6%)
-Limited sunlight exposure (Full time indoor occupation)	Vitamin D level (ng/ml)	6.9 (4.5–10.5)	6.8 (4.0–11.9)	6.5 (3.7–9.3) NS
-Lack of dietary vitamin D food intake	Vitamin D intake (μg)	3.3 ± 1.7	3.2 ± 1.8	3.1 ± 1.8 ^NS^
**Related disease?** *Correct answer:*	N (%)	105 (11.2%)	382 (40.6%)	453 (48.2%)
- Osteomalacia, rickets	Vitamin D level (ng/ml)	6.6 (4.4–13.5)	6.7 (4.1–10.0)	6.8 (3.8–9.9) ^NS^
-Osteoporosis and bone fractures	Vitamin D intake (μg)	3.3 ± 1.9	3.0 ± 1.7	3.2 ± 1.8 ^NS^
**Food sources of vitamin D** *Correct answer*:	N (%)	87 (9.3%)	369 (39.3%)	483 (51.4%)
- Sea food (fish, shrimps, tuna, etc.), egg yolk, liver -Dairy products (milk, cheese,	Vitamin D level (ng/ml)	6.6 (4.3–11.7)	6.6 (4.1–10.3)	6.8 (3.9–9.9) ^NS^
yogurt, etc. …), breakfast cereals	Vitamin D intake (μg)	3.2 ± 2.3	3.0 ± 1.8	3.3 ± 1.7 ^NS^
**Familiar with the term “osteoporosis” and preventive** **strategies** *Correct answer*:	N (%)	70 (7.5%)	169 (18.0%)	701 (74.5%)
- weakening of bones, which increases risk of fractures	Vitamin D level (ng/ml)	6.6 (4.5–12.7)	6.5 (4.0–9.3)	6.9 (3.9–10.2) ^NS^
- Prevented by eating calcium and vitamin D-rich foods, exercise	Vitamin D intake (μg)	3.2 ± 2.0	3.4 ± 2.2	3.1 ± 1.7 ^NS^

Serum vitamin D levels expressed as median (IQR) and compared in three groups by using Kruskal-Wallis test

Vitamin D intake expressed as mean ± SD and compared in three groups by using one-way ANOVA test. NS: Non-significant (p > 0.05)

* For each question, answer one item and both items considered as “Somewhat aware” and “Completely aware”, respectively

### Behavior toward sunlight and sunscreen usage of participant

**Fig. 1:**
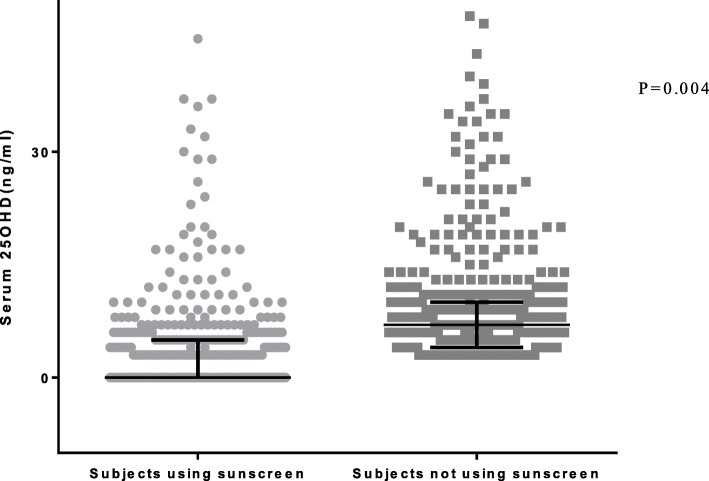
Individuals who used sunscreen compared to individuals who did not use sun screen are more likely to be deficient in Vit D. Median (interquartile range; IQR) of 25-hydroxyvitamin D levels were 6.1(3.7–9.2) ng/ml in sunscreen user individuals (*n* = 441), 7.3(4.4–10.7) ng/ml in non-sunscreen users(*n* = 449). Bars indicate IQR. *p* = 0.004; Mann-Whitney test

Girls who used sunscreen were more likely to be deficient in Vit D compared to individuals who did not use sunscreen (*P* = 0.004; Fig. 1). Approximately 30.42% of participants reported that they used sunscreens only in the morning, 13.72% twice a day, 2.76% three times a day and half of the participants 53.1% reported that they had never used sunscreens (Table 3). Among sunscreen users, levels of Vit D were not significantly associated with the frequency, amount, SPF, of the sunscreen used, nor the season and location (*P* > 0.05; Table 3).

**Table 3:** Sunscreen usage of participants (n = 441)

Question items	Response	Number (%)	Vitamin D level (ng/ml)	P value*
How often do you use sunscreens?	Only morning	286 (30.42)	6.2 (3.9–9.5)	0.96
	Twice a day	129 (13.72)	6.1 (3.1–9.1)	
	Thrice a day	26 (2.76)	6.9 (3.0–9.6)	
In which season do you use sunscreens?	Only summer	131 (13.93)	5.9 (3.7–90)	0.82
	Spring & summer	81 (8.61)	6.4 (3.8–10.0)	
	All season	230 (24.46)	6.2 (3.7–9.5)	
Sun protection factor (SPF)	< 30	82 (8.83)	6.7 (4.9–9.2)	0.11
	30–50	123 (13.08)	6.6 (4.1–10.1)	
	> 50	87 (9.25)	5.5 (3.0–9.4)	
	No idea	148 (15.74)	5.7 (3.4–9.0)	
What location do you use sunscreens?	Only face	314 (33.4)	6.2 (3.7–9.3)	0.73
	Face & hands	124 (13.19)	6.4 (4.0–9.2)	
	Most of the body	3 (0.31)	3.1 (3.0–29.7)	

* Serum vitamin D levels expressed as median (IQR) and compared by using Kruskal Wallis test

## Discussion

This is the first study to evaluate the knowledge, and some behaviors that affect Vit D status among female adolescents in northeastern Iran. We found that knowledge about Vit D in young women and girls from Mashhad and Sabzevar in Iran is very limited, and approximately half of the population did not know about the role of Vit D in health and disease. According to recent studies, Vit D deficiency has been shown in many Asian countries [35, 36]. The role of Vit D is especially important in adolescent girls; since Vit D status affected all aspects of growth, development and puberty in this age [37]. Our study suggests that a majority of our population may be deficient for Vit D.

In addition, few students were aware of the causes of Vit D deficiency (16.7%), the main sources of Vit D and the required time to spend outside to get adequate Vit D level (9.3%). This lack of knowledge about the sources of Vit D and causes of deficiency is a major concern. In various other studies, different levels of knowledge about Vit D have been reported. The majority of Indian (53.3%) students knew that sunlight is the main source of Vit D [26]. In one study among Omani female university students, about 90% of responders were aware of Vit D and sunlight as its most important source [38].

In a survey among private university students in Malaysia aged between 16 and 30 years old, only 7.2% of the respondents reported that they did not know from where Vit D is derived, whereas 69.2% of participants properly identified sunlight as an important contributor [39]. In all of these studies, participants were university students, studying at a tertiary level, and would therefore be expected to have a good knowledge of health issues compared to our sample population.

In a recent investigation among young student (aged 18–25 years) in Pakistan, only 9% of individuals were able to identify the correct food sources of Vit D, 33% were aware of the bone health advantages of Vit D and 36% knew that sunlight exposure was a major factor affecting Vit D synthesis in body [40]. Also, knowledge about Vit D, its sources and health benefits was very limited (28.8%) in Emirati and International tourist students in Dubai [41].

Another important finding of this study was that 46.9% of girls never used sunscreens, different to the findings within a study cohort in Hong Kong in which reported 44.4% of middle-aged and elderly women used a parasol to shade themselves from the sun, but the majority (81.2%) did not use sunscreen products [25]. However, two reports from Saudi Arabia and Vietnam reported a higher use of sunscreen particularly in females [42, 43].

Due to the sunny weather in most seasons of year in Iran; sunscreen usage is the most efficacious way for protection of the skin from sunlight over-exposure. We did not explore the reasons for this behavior, but it may be due to alterations in lifestyle because of the adoption of modern behavior norms, and an intention to avoid tanning (particularly females) are probable factors for the reduction in sun exposure and choosing sunscreen as a most effective approach of mitigating skin tanning in the female students.

In the present study, girls who used sunscreen compared to those who did not use sunscreen were more likely to have lower Vit D levels. In a recent systematic review of 76 studies, the evidence of experimental studies support the theoretical risk which sunscreen use may have on Vit D status, while the weight of findings from field trials and observational surveys indicates that the risk is low [44]. In the Muslim women, face and hands are the only body surfaces exposed to sun. So, sunscreen use and fully covering their body can put their health at risk. Al-Saleh and co-workers reported the high prevalence of Vit D among Saudi girls compared to boys due to the double negative effects of sunscreen using and clothed their body fully during sun exposure [45].

Our results did not show a significant association between the knowledge of the participants about Vit D and serum levels and dietary intake of Vit D. Similarly, no significant relationship was found between Vit D levels with nutritional status and daily living habits in private university students in Selangor and Saudi Arabia [46, 47]. Additionally, sun exposure over the previous week, sunscreen usage, oily fish consumption was not found to be associated with Vit D status among pregnant women [48]. As for our study and previous research, no statistically significant relationship was found between serum levels and dietary intake of Vit D with knowledge of Vit D, either before, or after adjustment for potential confounder in Saudi premenopausal women [49]. Interestingly, in a study including 1044 from the general population in Kuwait, 80% of people were found to have adequate knowledge; although Vit D deficiency remained prevalent among them [50]. In present study, the lack of association between knowledge and Vit D levels may be attributable to the high prevalence of Vit D deficiency in our population. Moreover, the less aware about Vit D food sources is also troubling among our participants.

Whilst knowledge and awareness play an important role in reducing the burden of any disorders, analysis of knowledge of Vit D suggests that doctors and health professionals must be especially careful in providing advice to people with these demographic characteristics. They require full information in order to make any lifestyle changes. The government and policy makers should consider how to improve the prevalence of Vit D deficiency through the use of mass media to promote awareness toward Vit D as well the need to test for Vit D and treat Vit D deficiency.

Our findings should be interpreted within the context of the study’s strengths and limitations. This study surveyed a large population of girl students. Notably, serum 25OHD concentrations were measured in all participants, and information regarding dietary Vit D were collected. This allowed the association between knowledge and behaviors with respect to Vit D could be explored. One limitation of this study was that all the participants were adolescent female, which limits the generalizability of the results to the general population. Moreover, we did not collect data regarding outdoor activity/frequency/duration; so its relationship with Vit D levels was not possible. Since the study was performed in the Iranian population, whose religion, lifestyle and cultural background may be different from other populations, the our findings may not be comparable to studies in other parts of the world.

## Conclusion

The majority of girls and young women possess inadequate knowledge about the major sources of Vit D, and its deficiency-related health disorders. However, surprisingly, the levels of serum and dietary intake of Vit D were not significantly different for those who had knowledge about the importance of Vit D consumption with those who did not. Public health education, such as workshops or training programs should be developed for the prevention of Vit D deficiency in the Iranian population especially in young girls who are more predisposed to Vit D deficiency.

## Supplementary Information

**Additional file 1: Supplementary File 1.** Questionnaire on knowledge regarding vitamin D and sunscreen use. The questionnaire comprised of three sections. Section 1 comprised questions on demographic information. Section 2 concerned questions about behavior towards the usage of sunscreen. Section 3 was designed to evaluate the knowledge of participants about vitamin D.

## Data Availability

The datasets used and/or analyzed during the current study are available from the corresponding author on reasonable request.

## Abbreviations

IQR: Interquartile range
FFQ: Food frequency questionnaire
NS: Non-significant
SPF: Sun protection factor
UV: Ultraviolet
Vit D: Vitamin D
25OHD: 25-hydroxy vitamin D
