# Supplementary material for: Evaluation of the knowledge regarding vitamin D, and sunscreen use of female adolescents in Iran
Source: BMC Public Health. 2021 Nov 10;21:2059. doi: 10.1186/s12889-021-12133-5 (PMC8579675; doi:10.1186/s12889-021-12133-5)
Supplement: Supplementary file 1 — Additional file 1: Supplementary File 1. Questionnaire on knowledge regarding vitamin D and sunscreen use. The questionnaire comprised of three sections. Section 1 comprised questions on demographic information. Section 2 concerned questions about behavior towards the usage of sunscreen. Section 3 was designed to evaluate the knowledge of participants about vitamin D. [file 12889_2021_12133_MOESM1_ESM.docx]

**Section 1**

1. How old are you?
2. Number of family members?
3. 2-4 b) 5-7 c) 8-10 d) more than 11
4. With whom do you live؟
5. Father & mother b) Father c) mother d) Other family
6. Do you have a room for your sole use?
7. Yes b) No
8. Are one or both of your parents dead?
9. Yes b) No
10. Are the parents divorced?
11. Yes b) No

1. What is your father’s occupation?

a) Worker b) Employee c) Tradesmen market d) Religious e) Other f) Deceased

1. What is your mother occupation?

a) Worker b) Employee c) Housewife d) Other e) Deceased

1. What is your father’s educational attainment (years)?

a) 0-9 b) 10-13 c) ≥13

1. What is your mother’s educational attainment (year)?

a) 0-9 b) 10-13 c) ≥13

**Section 2**

1. How often do you use sunscreens?

a) Once a day b) Twice a day c) Thrice a day

1. In which season do you use sunscreens?

a) Only summer b) Spring & summer c) All year

1. What is the sun protection factor (SPF) do you use?

a) Less than 30 b) 30-50 c) +50 d) No idea

1. Where do you use sunscreens?

a) Only on my face b) On my face & hands c) Over most of my body

**Section 3**

1. Do you know the roles and benefits of vitamin D in the body?
2. What do you think are the causes of vitamin D deficiency?
3. What are some specific diseases/symptoms related to vitamin D deficiency?
4. What are the sources of vitamin D in the diet?
5. Do you know about term “osteoporosis”? Can you suggest a suitable strategy for prevention of vitamin D deficiency?
